# Microbial profiles at baseline and not the use of antibiotics determine the clinical outcome of the treatment of chronic periodontitis

**DOI:** 10.1038/srep20205

**Published:** 2016-02-01

**Authors:** S. Bizzarro, M. L. Laine, M. J. Buijs, B. W. Brandt, W. Crielaard, B. G. Loos, E. Zaura

**Affiliations:** 1 Department of Periodontology Academic Centre for Dentistry Amsterdam (ACTA), University of Amsterdam and VU University Amsterdam, The Netherlands; 2 Department of Preventive Dentistry Academic Centre for Dentistry Amsterdam (ACTA), University of Amsterdam and VU University Amsterdam, The Netherlands.

## Abstract

Antibiotics are often used in the treatment of chronic periodontitis, which is a major cause of tooth loss. However, evidence in favour of a microbial indication for the prescription of antibiotics is lacking, which may increase the risk of the possible indiscriminate use of antibiotics, and consequent, microbial resistance. Here, using an open-ended technique, we report the changes in the subgingival microbiome up to one year post-treatment of patients treated with basic periodontal therapy with or without antibiotics. Antibiotics resulted in a greater influence on the microbiome 3 months after therapy, but this difference disappeared at 6 months. Greater microbial diversity, specific taxa and certain microbial co-occurrences at baseline and not the use of antibiotics predicted better clinical treatment outcomes. Our results demonstrate the predictive value of specific subgingival bacterial profiles for the decision to prescribe antibiotics in the treatment of periodontitis, but they also indicate the need for alternative therapies based on ecological approaches.

The oral microbiome is an extremely dynamic environment. Due to the anatomical characteristics of the oral cavity, a large variety of microbes have the opportunity to nest and create stable biofilms. The complex equilibrium between commensal, opportunistic and pathogenic species is responsible for the maintenance of a healthy state (symbiosis) or the onset of infectious diseases (dysbiosis). As an open environment, the oral cavity is continuously exposed to a number of external factors that can influence the dynamics of the oral microbiome. Consequently, the treatment of oral infections can be difficult.

Among oral diseases, periodontitis, especially the chronic form of the disease, represents one of the most important causes of tooth loss in the adult population^1^. Periodontitis is an oral inflammatory disease of the supporting tissues of the teeth that is caused by aberrant immune reactions to bacteria that colonize the dental root surface. When left untreated, in susceptible individuals, the inflammatory infiltrate can increase and cause the destruction of the periodontal tissues. The results are the loss of connective tissue attachment, alveolar bone and eventually in severe cases, exfoliation of the tooth^2^. The most frequently occurring forms of periodontitis are chronic and aggressive^3^. Aggressive periodontitis is characterized by an usually early age of onset (<25 years old) and a relatively high progression rate of periodontal tissue loss, but the prevalence of this condition is relatively low, 0.1–5%^4^,^5^. Chronic periodontitis mainly affects adults and is characterized by a slow rate progression of periodontal tissue loss, but its prevalence is much higher and varies between 6–30% depending on the country and characteristics of the population under investigation^6^,^7^,^8^.

Microbiological investigations have identified the taxonomic communities that are primarily associated with a disease state. The genera *Aggregatibacter, Porphyromonas, Prevotella*, and *Tannerella*^9^ have traditionally been the most strongly associated taxa with this disease, but more recently, other taxa, such as *Synergistetes* and *Filifactor*, have also been associated with periodontitis^10^.

Therapy for periodontitis aims to reduce the total bacterial load and to suppress pathogenic species in the subgingival microbiome via non-surgical mechanical disruption and removal of the subgingival biofilm. The ultimate goal is to re-establish a microbiome that is compatible with health^11^,^12^,^13^. However, the recurrence of inflammation and progression of periodontal breakdown is common^14^,^15^. The adjunctive use of systemic antibiotics, particularly the combination of amoxicillin and metronidazole, has been found to enhance the resolution of periodontal inflammation compared with basic periodontal therapy alone in some studies^16^ and has also been found to be more effective in reducing bacterial species traditionally associated with periodontitis^17^,^18^,^19^. However, not all patients benefit from antibiotics^18^. The presence of specific taxa before and after therapy has not been consistently demonstrated to have a high predictive value, neither as an indicator of the progression of the disease nor as an indicator of the prescription of antibiotics^20^. Consequently, the choice of the use of antibiotics is still based on clinical indicators of inflammation and primarily on the experience of the clinician^21^. This leads to a high risk for the inappropriate prescription of antibiotics in the treatment of chronic periodontitis and an increased risk of the development of antibiotic resistance. Therefore, there is a need to deeply investigate the changes in the subgingival microbiome following the use of antibiotics to understand the conditions in which periodontitis-associated biofilms are more susceptible to the use of amoxicillin and metronidazole.

The effects of the use of antibiotics together with non-surgical periodontal therapy on the composition of the subgingival microbiome has been widely investigated with targeted techniques such as anaerobic culturing^19^, DNA hybridization^18^,^22^, polymerase chain reaction^17^,^20^ and micro-arrays^23^. However, these techniques are not able to investigate the composition of the microbial community in the manner that like open-ended techniques, such as 16S rDNA amplicon sequencing, are able to investigate^10^,^24^. To date, only case reports have described the effects of antibiotics on subgingival communities in periodontitis patients^25^,^26^,^27^.

Therefore, the primary aim of this one-year follow-up study was to describe, using 16S rDNA amplicon sequencing, the effect of basic periodontal treatment with or without the use of systemic antibiotics, on the subgingival microbiome compositions in a population affected by chronic periodontitis. Thereafter, we explored the microbial community compositions in relation to the clinical outcomes.

## Results

We investigated the subgingival microbiome of 37 subjects affected by chronic periodontitis who received standard non-surgical periodontal therapy (scaling and root planing) and chlorhexidine rinses. Additionally, 18 of the 37 subjects (Test) received systemic antibiotics (combinations of amoxicillin and metronidazole), which are currently considered the most effective antimicrobial treatment for periodontitis^28^. The groups did not differ in terms of age, gender, ethnicity, smoking or education level (Table S1). Bacterial plaque from subgingival lesions was sampled at baseline and this sampling was repeated at 3, 6 and 12 months after therapy.

In both the Test and Control groups, the periodontal therapy resulted in improvements in all of the clinical parameters (Table S2). In the Test group, the sample sites exhibited larger reductions in probing pocket depth but not in clinical attachment level, bleeding on probing or the presence of plaque (Table 1). At the full mouth level, there was a significantly greater reduction only in the number of deep pockets (>7 mm) in the Test group, and no differences were observed in the other clinical parameters.

### Sequencing results

Of the 148 samples, one sample from the Test group (month 12) did not yield sufficient high quality DNA. Of the 1.5 million sequencing reads, 90.7% passed the quality filter and 83% remained after the removal of chimeric sequences. Two samples from the Test group (one at month 3 and one at month 6) yielded number of reads after quality filtering that were too low (29 and 449, respectively) and were excluded from further analyses. The remaining 145 samples exhibited an average of 8783 reads/sample (SD 5710, range 1406–31831 reads). The reads were clustered into 624 operational taxonomic units (OTUs). To allow between-sample comparisons, the data were randomly subsampled at 1400 reads/sample, which reduced the number of OTUs to 472. These OTUs were classified into 13 bacterial phyla or candidate divisions and into 195 genera or higher taxa (Table S3).

### Microbiological results after therapy (genus level)

**Figure 1:**
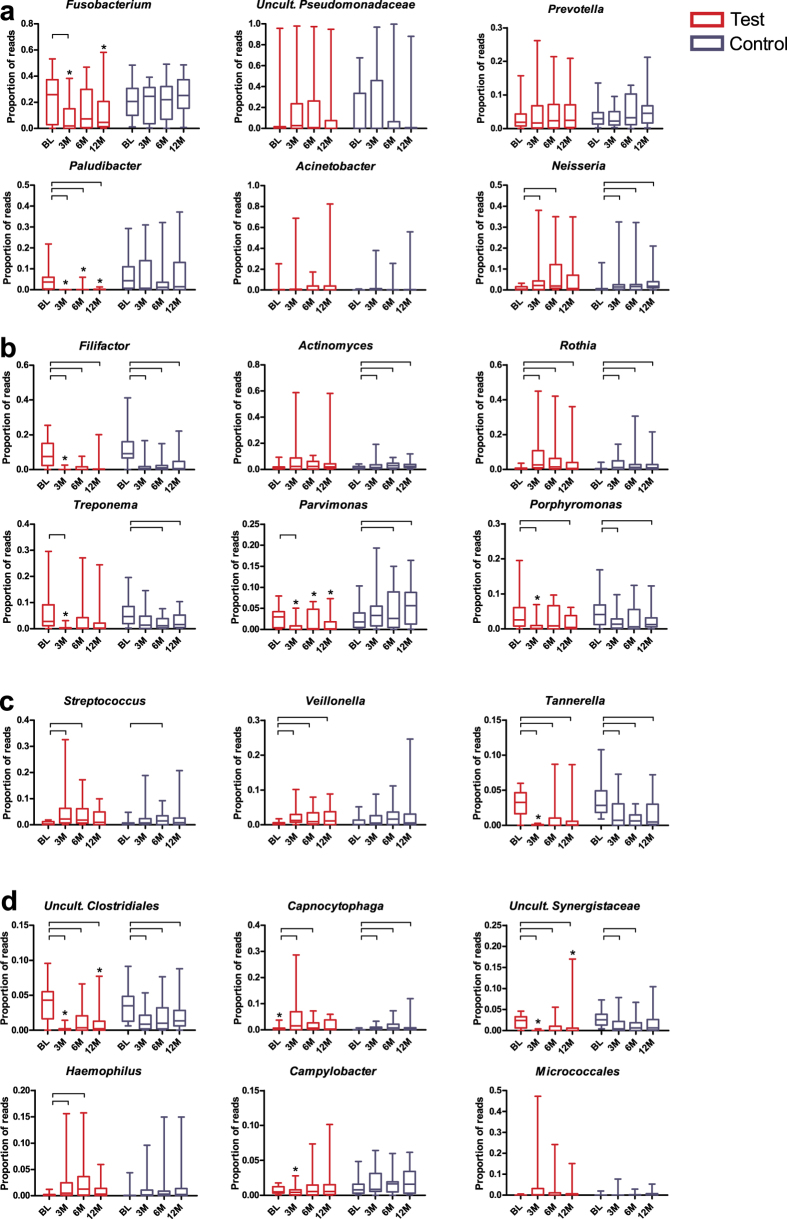
Relative abundances of the major genera or higher taxa. (**a**) Taxa that were significantly reduced by both treatments; (**b**) taxa that increased after the treatments; (**c**) taxa that were affected only in the Test group and (**d**) that were minimally affected by any of the treatments, for each time point (BL – baseline, 3M – 3 months, 6M – 6 months, 12M – 12 months) and treatment (Test or Control). *Statistically significant difference between the treatment groups (*P* < 0.05; Mann-Whitney test). Connectors connect the time-points within the same group in which the taxa proportions differed significantly (*P* < 0.05, Wilcoxon Signed Ranks test).

First, we analysed single microbial taxa (Fig. 1). Among the major genera, no differences were observed between the Test and Control groups at baseline, except for the genus *Capnocytophaga*, which was higher in the Test group (*P* = 0.031). When we assessed the effects of the treatments on the microbial compositions at genus or higher-taxon level, we found that both treatments resulted in significant reductions of the proportions of the genera *Filifactor*, *Tannerella* and uncultured *Clostridiales* family xiii *incertae sedis* (*P* < 0.05) compared with baseline (Fig. 1); these reductions remained significant up until the 1-year follow-up (Fig. 1A). Additionally, the proportions of the genera *Porphyromonas* and *Treponema* and uncultured *Synergistaceae* were significantly reduced in both treatment groups, although these reductions were not apparent at all time-points (Fig. 1A). The reductions in these taxa were significantly greater at 3 months in the Test group compared with the Control group (*P* < 0.05) and in the uncultured *Clostridiales* and *Synergistaceae* at the 1-year follow-up. Six of the major genera exhibited post-therapy increase in proportions (Fig. 1B). *Neisseria*, *Rothia*, *Capnocytophaga* and *Streptococcus* increased in both groups compared with baseline, while proportions of *Veillonella* and *Haemophilus* increased significantly only after antibiotic exposure. Slight but significant increases were observed in the Control group in *Parvimonas* (Fig. 1C) and *Actinomyces* (Fig. 1D). *Paludibacter*, *Fusobacterium* and *Parvimonas* were significantly reduced only in the Test group (Fig. 1C). *Paludibacter* remained significantly reduced until the 1-year follow-up. Among the dominant taxa, none of the treatments significantly affected the proportions of the genera *Prevotella*, *Acinetobacter*, or uncultured *Pseudomonadaceae* or the order Micrococcales (Fig. 1D).

### Microbial diversity and similarity after therapy

There were no significant differences in microbiome diversities (Shannon diversity index) between the two treatment groups at any of the time points, and none of the treatment groups exhibited significant changes in diversity over time (Fig. S1).

**Figure 2:**
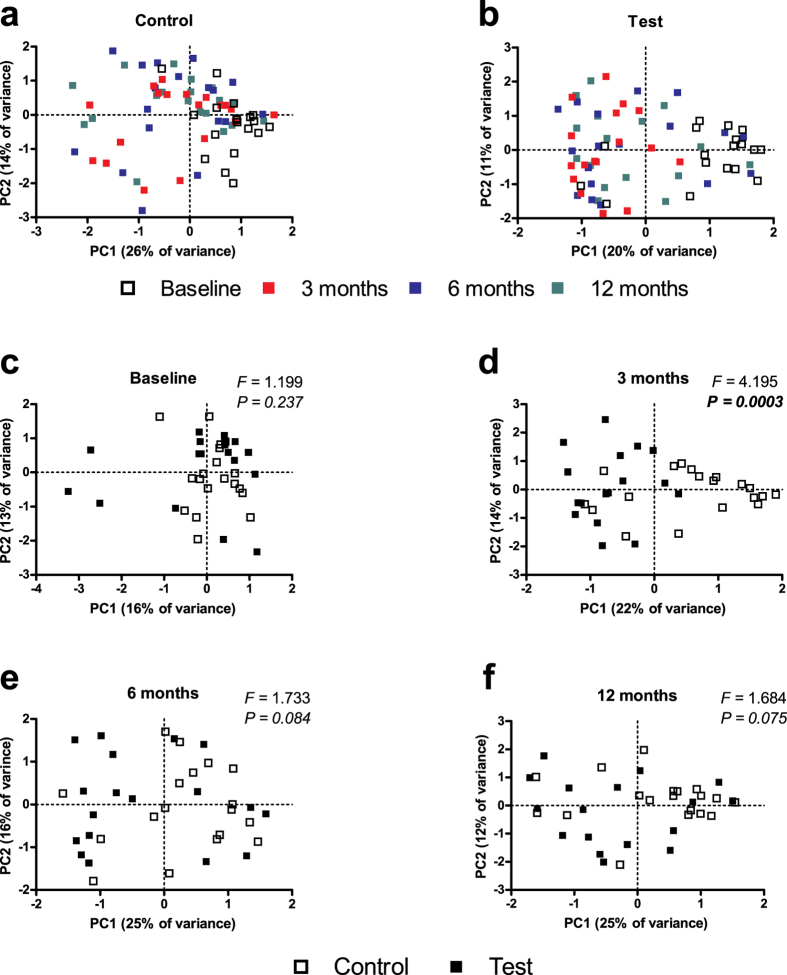
Principal component analysis (PCA) plots. Plots represent the samples in the Control group (**a**) and the Test group (**b**), coloured by time-point (each colour indicates a different time-point). In panels (**c–f**) plots represent the individual samples for each separate time-point. White squares represent individuals in the control group, black squares represent individuals in the test group (F and *P* values were obtained with PERMANOVA test).

PCA was performed to ordinate the multivariate data. Irrespective of the group, the baseline samples clustered together (Fig. 2A,B). Both treatment groups exhibited significant differences in microbiome profiles between the baseline and the follow-up visits (F = 2.8, *P* < 0.0003 and F = 2.2, *P* < 0.001 for the Test and Control groups, respectively). In the Test group, the largest difference was observed between the baseline and 3 months follow-up (F = 7.3, *P* = 0.0006), followed by the 6-month (F = 4.5, *P* = 0.007) and the 12-month follow-up (F = 3.5, *P* = 0.015). In the Control group, all three visits exhibited comparable difference with the baseline visit (F = 4.3, *P* = 0.0006; F = 3.9, *P* = 0.002; F = 4.3, *P* = 0.0006 between the baseline and the 3-month, 6-month or 12-month follow-up, respectively). The microbiome profiles of the two groups differed significantly only at the 3-month visit (F = 4.195, *P* = 0.0003, Fig. 2D), and not at the baseline (Fig. 2C), 6-month (Fig. 2E) or 12-month (Fig. 2F) visits. These findings indicate that the significantly greater influence of antibiotics on the microbiome observed at three months was not retained in the longer term.

**Figure 3:**
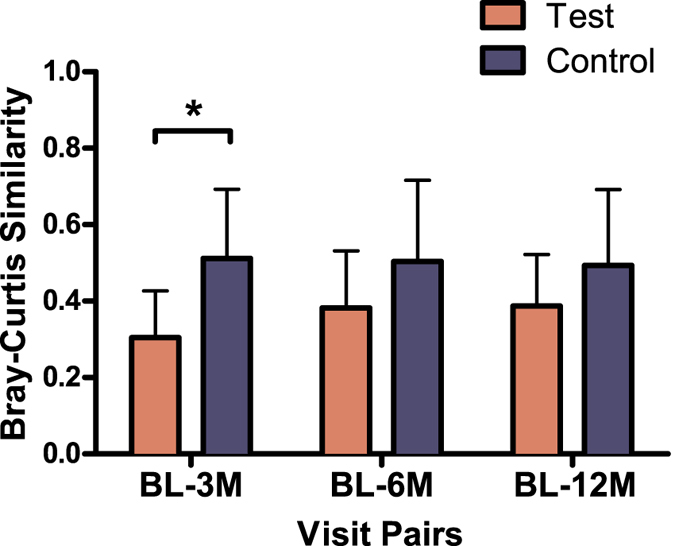
Average Bray-Curtis similarity of microbiome profiles. Bars represent differences between the baseline visit (BL) and the other visits (3M – 3 months, 6M – 6 months, 12M – 12 months after therapy) for each individual according to treatment group (Test and Control). Connector indicates statistically significant difference between the groups (*P* < 0.001, independent samples T-test).

Theses results were confirmed by the analysis of the effects of the two treatments on the magnitudes of the microbial shifts. We assessed these effects by comparing the microbiome profiles of the samples at the baseline with those collected at the other time points from the respective individuals using the Bray-Curtis similarity index (Fig. 3). The lowest similarity was observed between the baseline and the 3-month post-treatment samples in the Test group, whereas the Control group samples exhibited greater similarity between the baseline and the respective post-treatment samples. These differences were statistically significant between the baseline and the 3-month samples (Fig. 3). Thus, the greatest shift in microbiome composition was observed at 3 months in the Test group.

### Microbiological shift in relation to treatment outcome

**Figure 4:**
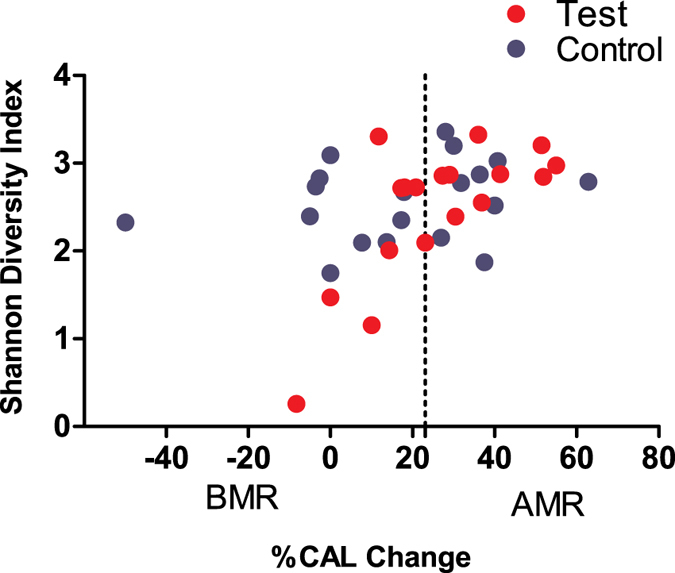
Correlation between Shannon diversity index at baseline and per cent change of Clinical Attachment Level (% CAL change) of the sampled sites at 12 months. Light coloured dots represent patients in the test group and dark coloured dots represent patients in the control group. The vertical line represents the cut-off point (24.08%) between Below Median Responders (BMRs) and Above Median Responders (AMRs).

**Figure 5:**
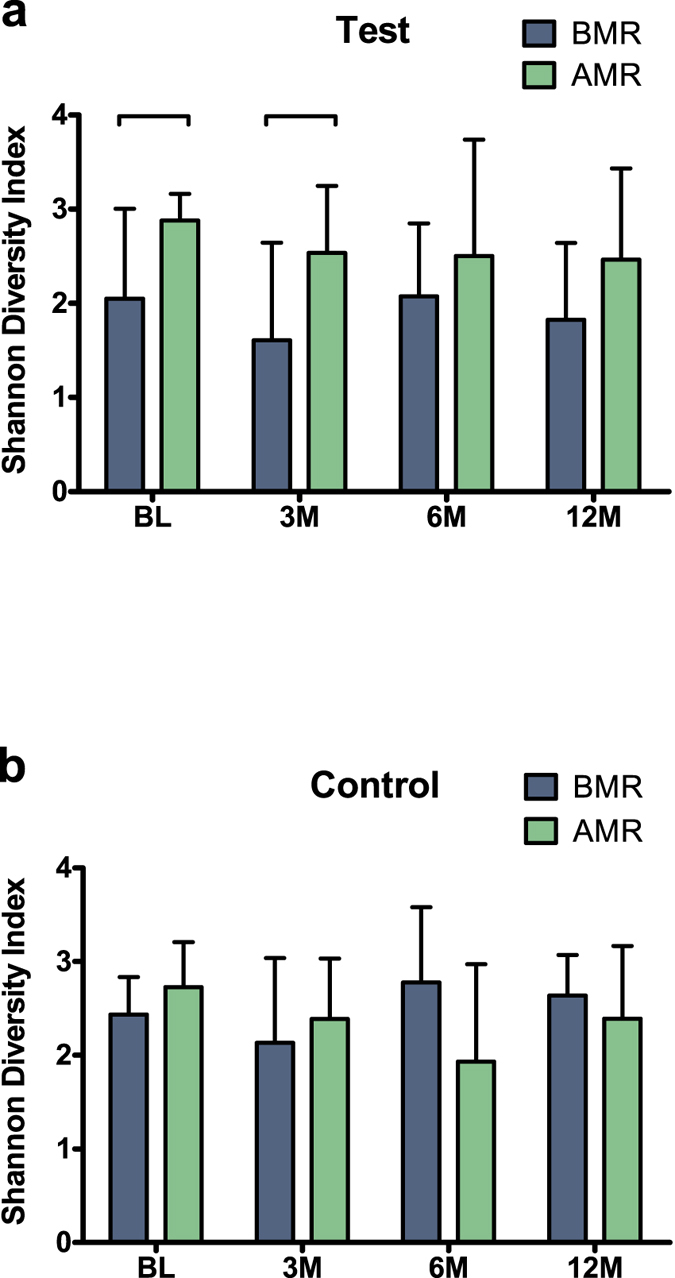
Average Shannon Diversity Index according to treatment modality and treatment outcome. (**a**) Below Median Responders (BMRs) and Above Median Responders (AMRs) in the Control group and (**b**) the Test group samples per time point (BL – baseline, 3M – 3 months, 6M – 6 months, 12M – 12 months). The error bars indicate standard deviation. The connector indicates a statistically significant difference (*P* < 0.05, Independent samples T-test).

Based on the clinical response to treatment we subdivided all patients into two groups, i.e. those who exhibited better responses to treatment (above median responders = AMRs) and those who presented poorer responses (below median responders = BMRs). We identified 19 BMRs and 18 AMRs. The validity of this subdivision was confirmed by the clinical findings after treatment (Table S4). This subdivision led to comparable distributions of patients who were treated with antibiotics between these groups; indeed, 50% of the patients in the Test group (N = 9) and 52% of the patients in the Control group (N = 10) belonged to the BMR group. Interestingly, 73.7% of the BMR patients were smokers, and nearly half of these patients (42.9%) were treated with antibiotics, whereas only 7 of 18 AMR patients (38.9%) were smokers. We assessed the relationship of the clinical responses of all subjects with the microbial diversity at baseline and at the different follow-up visits. We found that the clinical improvement, represented by the per cent gains in clinical attachment level (CAL) at 12 months, was significantly positively correlated with the microbiome diversity at baseline (r = 0.469; *P* = 0.003, Fig. 4). In the Test group, the BMRs exhibited significantly lower microbiome diversity at baseline and at the 3-month visit compared with the AMRs (Fig. 5A). In the Control group no difference between the BMRs and AMRs regarding microbial diversity was found at all any of the time points (Fig. 5B).

**Figure 6:**
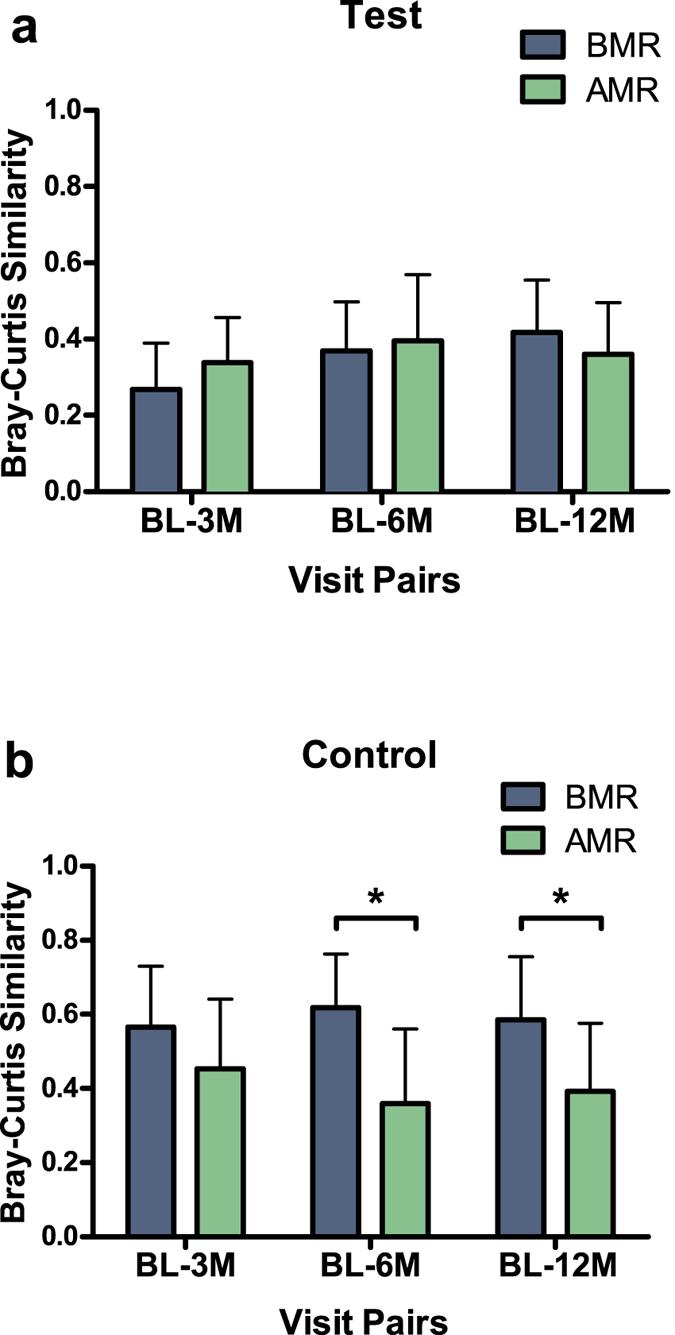
Average Bray-Curtis similarity in microbiome profiles according to treatment modality and treatment outcome. Bars represent differences between the baseline visit (BL) and the other visits (3M – 3 months, 6M – 6 months, 12M – 12 months since the treatment) of each individual by clinical response at one-year follow-up (**a**) in the Test group and (**b**) in the Control group. Test: *N* = 9 Above Median Responders (AMRs), *N* = 9 Below Median Responders (BMRs); Control group: *N* = 10 Above Median Responders (AMRs), *N* = 9 Below Median Responders (BMRs). Error bars indicate standard deviations. Connector indicates a statistically significant difference between the groups (independent samples T-test).

Thereafter, we assessed whether the clinical response could be related to changes in the microbiome profile. The Bray-Curtis similarity between microbiome profiles of the baseline and follow-up data from the same individuals did not differ between the AMRs and BMRs in the Test group (Fig. 6A). In the Control group, the microbiome similarity between baseline-6 months and baseline-12 months groups were significantly lower among the AMR patients than the BMR patients (*P* < 0.05, Fig. 6B). In other words, in the Control group, the AMRs could be discriminated from the BMRs by the greater shift in microbiome composition over time compared with the baseline samples.

### Association of microbial taxa with treatment outcome

We explored the possible associations between the treatment outcome and the presence of specific taxa at baseline and at the follow-up time points. The baseline AMR samples had a significantly higher proportion of *Porphyromonas* (*P* = 0.003), *Treponema* (*P* = 0.034), uncultured *Clostridiales* family xiii *incertae sedis* (*P* = 0.025) and *Filifactor* (*P* = 0.042) regardless of Test or Control group membership. At three months, the AMRs exhibited significantly higher proportions of *Actinomyces* (*P* = 0.004), *Streptococcus* (*P* = 0.001), *Veillonella* (*P* = 0.034), *Neisseria* (*P* = 0.04) and *Haemophilus* (*P* < 0.0001). At six months, no genera differed according to response, but at 12 months the genus *Tannerella* was significantly lower in the AMRs (*P* = 0.024).

When we compared the AMRs and BMRs by treatment group, we found higher proportion of *Porphyromonas* (*P* = 0.011) in the samples of the AMR in Test patients at baseline and no difference for any genera between the AMRs and BMRs in the Control group. At three months, the AMRs of the Test group had significantly more *Actinomyces* (*P* = 0.011) and *Haemophilus* (*P* = 0.027), whereas in the Control group, in addition to *Haemophilus* (*P* < 0.0001), also *Rothia* (*P* = 0.043) and *Streptococcus* (*P* < 0.0001) were significantly more abundant in the AMRs. At 6 months, significant differences were found only in the Control group; AMRs had lower proportions of *Paludibacter* (*P* = 0.034), *Tannerella* (*P* = 0.009), *Clostridiales* family xiii *incertae sedis* (*P* = 0.003), *Treponema* (*P* = 0.034), uncultured *Synergistaceae* (*P* = 0.004) and *Filifactor* (*P* = 0.012). At 12 months, the AMRs from the Test group had a higher proportion of *Capnocytophaga* (*P* = 0.027), while the Control group exhibited more *Rothia* (*P* = 0.008) and less *Paludibacter* (*P* = 0.043), *Tannerella* (*P* = 0.004) and uncultured *Synergistaceae* (*P* = 0.043) than the BMRs (Table S5).

At OTU level at baseline, in the Test group samples OTU311 (uncultured *Pseudomonadaceae*, BLAST search results: 100% ID *Pseudomonas fragi*) was negatively correlated (r = −0.659, *P* = 0.003), whereas the following other OTUs were positively correlated with clinical improvement: OTU177 (*Porphyromonas*, BLAST: 100% ID with uncultured bacterium clone ncd2738e09c1, r = 0.619, *P* = 0.006), OTU420 (uncultured *Clostridiales* family xiii *incertae sedis*, BLAST: 100% ID *Eubacterium nodatum*, r = 0.689, *P* = 0.002), OTU555 (*Filifactor*, BLAST: 100% ID *Filifactor alocis*, r = 0.612, *P* = 0.007) and OTU385 (*Tannerella*, BLAST: 100% ID *Tannerella forsythia*, r = 0.503, *P* = 0.033) (Fig. S2). In the Control group, none of the OTUs in the baseline samples exhibited any association with the treatment outcome.

### Microbial co-occurrence in relation to treatment outcome

**Figure 7:**
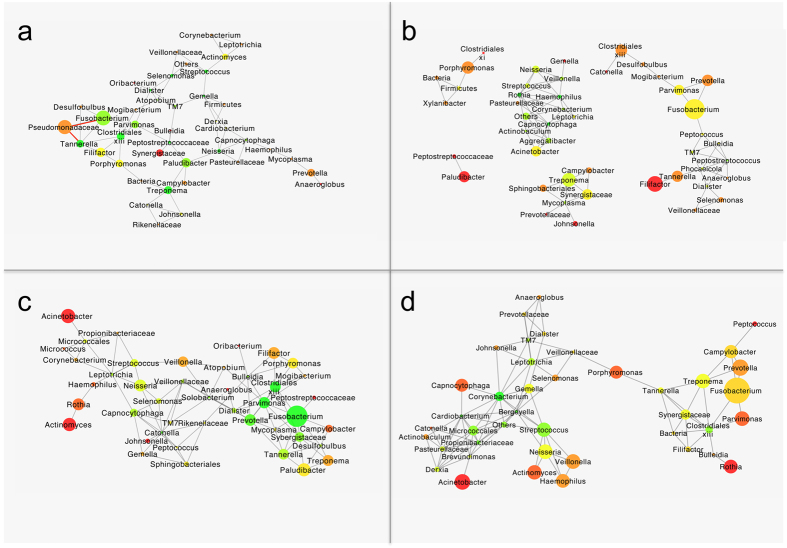
Microbial co-occurrence or mutual exclusion networks between genera. (**a**) Baseline taxonomic networks for the below median responders (BMRs) and (**b**) the above median responders (AMRs). (**c**) 12-month follow-up taxonomic network for below the median responders (BMRs) and (**d**) the above median responders (AMRs). Only edges that were significant by at least two of the methods (i.e. Pearson and Spearman correlations, Bray-Curtis similarity and Kullback-Leibler divergence) after correction for multiple comparisons were included in the network. The grey edges indicate positive correlations, and the negative correlations are indicated in red (*P* < 0.05). The size of the node is indicative of the relative abundance of the respective taxon. The colour of the node indicates the number of connections in the range of red to green; red indicates one connection, while green indicates >3 connections.

Members of microbial communities are known to form complex interactions. To investigate these interactions, we assessed the co-occurrence or mutual exclusion of microbial taxa at baseline and after one year based on the responses to treatment. At baseline, among the BMRs, we observed a fully interconnected topology of the genera associated with disease state (*Fusobacterium, Parvimonas, Clostidiales xiii* and *Tannerella* on the one side and *Paludibacter* and *Treponema* on the other side), which were preponderant and highly connected. These findings contrasted with those for the health-associated genera, which were weakly interconnected with each other and present in low abundance (Fig. 7A). Interestingly, in this network only mutually exclusive associations were found between the non-oral family *Pseudomonadaceae* and the families *Fusobacterium*, *Pseudomonadaceae* and *Tannerella* (Fig. 7A). In other words, these taxa are not likely to occur together in the same sample.

The baseline samples of the AMR group revealed a fully connected network of the health-associated genera including *Streptococcus, Neisseria, Rothia, Haemophilus* and *Veillonella* (Fig. 7B), whereas the disease-associated genera formed 4 disconnected subnetworks. *Tannerella, Filifactor, Prevotella*, *Fusobacterium* and *Parvimonas*, on the one side and *Treponema, Synergistaceae, Campylobacter* and *Sphingobacteriales* on the other side were predominant in two major networks, while *Paludibacter* and *Porphyromonas* were central in two other minor subnetworks (Fig. 7B).

One year after the therapy, the co-occurrence network of the taxa in the subgingival microbiomes of the BMRs was similar to the network observed at baseline in that the disease-associated genera were highly interconnected, with the exception that the health-associated genera (i.e. *Neisseria, Streptococcus* and *Capnocytophaga)* had increased in abundance (Fig. 7C). Among the AMR samples, of the 4 distinct networks at baseline, one network with two clearly distinct parts interconnected by *Porphyromonas* was apparent at the 12-month visit (Fig. 7D), i.e., a larger subnetwork with strong interconnections between relatively abundant health-associated genera and a smaller group of disease-associated genera in which *Clostridiales xiii* occupied a central role (Fig. 7D).

## Discussion

This study investigated the effect of non-surgical periodontal therapy with or without antibiotic use on the subgingival microbiome. Our results indicated that the characteristics of the subgingival microbiome at baseline and not the use of antibiotics predicted the long-term (12 months) clinical outcomes of the treatment of chronic periodontitis. This study is the first investigation that applied deep sequencing to patients in a randomized controlled trial who had or had not received adjunctive antibiotics, excluding previous case reports and cohorts treated only with non-surgical periodontal therapy^25^,^26^,^27^.

The periodontal therapies applied to the current population resulted both in a decrease in periodontal pathogenic taxa (*Filifactor, Tannerella, Synergistaceae, Porphyromonas and Treponema*) and an increase in oral health-associated bacteria. Regardless of the use of antibiotics, the patients exhibited a large variety in the individual microbial shifts in response to treatment without any distinct clustering of patients in our principal component analysis (PCA) after the therapies. Similar findings have been described by Schwarzberg and co-workers in patients after non-surgical periodontal treatment^27^. In our study, antibiotics had a significant adjunctive influence on the microbiome compositions only at 3 months and not at the 6- and 12-month visits. These findings indicate that the initially substantial influence of antibiotics on the subgingival microbiome diminishes over the long term. Our findings confirm the results of other similar investigations with comparable follow-up times, which have employed targeted techniques of bacterial detection^18^,^29^.

Previous studies that have used targeted microbial techniques have demonstrated that the presence of specific bacteria before treatment was poorly predictive of the indication for the adjunct use of antibiotics^20^,^30^,^31^. However, focusing on specific species and without considering the correlation between different communities can be insufficient to explain the dynamic change in biofilms in response to therapy. Although we are aware of the explorative nature of the current study as reflected in the low number of subjects in the subgroup analyses, we addressed the question of whether microbiological profiles correlate with the clinical responses to the treatment (AMRs and BMRs). In our population there were equal distributions of patients who were adjunctively treated with antibiotics in both response groups, which is surprising because we expected to observe a greater number of patients who had been treated with antibiotics in the group that exhibited better response (AMR)^18^,^28^. In contrast, a better clinical outcome at 12 months correlated with a more diverse microbiome at baseline, which suggests that the subgingival microbiome composition at baseline can be predictive of the future response to therapy. Lower diversity could indicate the presence of a stable and pathogenic biofilm, which is more difficult to eradicate even with the use of antibiotics due to the presence of some dominant (pathogenic) bacterial species.

When we accounted for specific bacteria, we found that the successful reduction of pathogenic taxa (i.e. *Porphyromonas, Clostridiales, Filifactor, Tannerella* and *Fusobacterium*)^10^ and an increase in health-associated bacteria were related to a clinical response above the median of the whole population. In the control group, the post-therapeutic persistences of *Filifactor, Synergistaceae, Treponema* and *Paludibacter*, which exhibited to decrease only in those who received antibiotic therapy, were associated with poorer responses.

In the Test group, poorer responses were associated with higher proportions of *Pseudomonadaceae* at baseline. *Pseudomonas* species and particularly *Pseudomonas aeruginosa* have recently been associated with periodontitis^32^ and peri-implantitis^33^ and *Pseudomonas* species have been reported to be difficult to eradicate with antibiotics^34^,^35^,^36^,^37^.

In general, we can extrapolate from our results that patients with highly diverse subgingival microbiome comprised mainly of oral taxa traditionally associated with periodontitis may have good prognoses for treatment outcomes with non-surgical periodontal therapy alone. The persistent presence of taxa after non-surgical treatment in poor responders, such as *Filifactor, Synergistaceae, Treponema* and *Paludibacter*, deserves more attention in further research. Patients with low microbial diversity and predominance of non-oral taxa such as *Pseudomonas* seem to respond less to non-surgical periodontal therapy regardless the use of antibiotics. For these patients, other antimicrobials or alternative treatments (e.g., probiotics and quorum sensing inhibitors) may be considered for further research^38^,^39^.

To better understand the complex interactions between the different bacteria, we investigated the co-occurrence networks of the different genera in relation to the clinical outcome. It has been demonstrated that the co-occurrence of specific microorganisms can be useful in predicting the resolution of diseased sites after therapy^40^. Our results confirm findings from previous studies that have demonstrated that both the presence of microorganisms, and the correlations between microorganisms are important for the efficacy of a treatment^40^,^41^. These findings are important to future research directions in terms of understanding the dynamics of the subgingival biofilm, understanding the mechanisms that are responsible for the shift of the subgingival biofilm from a symbiotic state to a dysbiotic state, and determining the diagnostic validity of specific bacterial correlations. In our study, we found that the baseline presence of health-associated bacteria with well-interconnected network could better predict the resolution of the periodontal inflammation and an improved clinical outcome. Whether the use of broad-spectrum antibiotics might be a double-edged knife in the treatment of periodontitis is an issue worthy of discussion; such antibiotics will suppress also health-associated species and may thus enhance the dysbiotic status of the oral microbiome. Indeed, relations between dysbiotic shifts of the gut microbiome following the use of different antibiotics and systemic diseases, such as colitis, inflammatory bowel disease and rheumatoid arthritis have been reported^42^.

The role of smoking on the clinical outcomes of our population also requires discussion. As previously mentioned, 42.9% of the BMR patients were treated with antibiotics. Notably, 73.7% of the BMR patients were smokers, whereas only 7 of the 18 AMR patients (38.9%) were smokers. This finding suggests that smoking probably has a larger influence on the subgingival microbiome than the use of systemic antibiotics and that the microbiomes of smokers do not react to therapy in the same manner in all individuals. These findings corroborate our previous investigation in which we reported a large variability in the microbiomes of periodontitis patients who smoke^24^.

On a critical note, although 16S rRNA gene amplicon sequencing is considered an untargeted method, there are no ‘universal’ primers that would amplify all bacterial taxa^43^. In this and in our previous studies^24^,^44^,^45^ we have used V5-V7 hypervariable region of the 16S rRNA gene. *In silico*, these primers amplify a broad spectrum of bacteria and cover 68% of the bacterial sequences deposited in the SILVA database^43^. However, these primers target no archaeal sequences. Archaea, especially *Methanobrevibacter oralis* are frequently found in periodontal pockets of periodontitis patients^46^. Therefore, we cannot exclude the role of these microorganisms in the subgingival microbial communities of our study population. To address this question, the use of other primers, for example, those targeting V4 of 16S rRNA gene or other approaches such as culturing^47^ or targeting using specific PCR probes^46^ would be required.

In conclusion, our results demonstrated that specific characteristics of the subgingival microbiome can be predictive of treatment outcomes. Furthermore, due to the complex interactions between the different microbial species, we suggest that future research should focus on new therapies that are based on ecological approaches. However, due to the large compositional differences in the oral microbiome between different individuals and the limited number of patients in the current study, the predictive value of our findings need to be confirmed by replicating these analyses in larger scale trials.

## Materials and Methods

### Study population

The patients were selected from a larger single-blinded (examiner/therapist), randomized, controlled clinical trial, that was performed from 2008 to 2014. Inclusion was based on the possibility to analyse a maximum of 150 samples excluding the necessary control samples. Thus, the first 37 consecutive patients who completed the trial were included. A periodontal case was defined if the presence of proximal attachment loss of ≥3 mm at ≥2 non-adjacent teeth could be assessed^48^. Smokers were defined by current smoking or quitting no longer than 6 months before the baseline visit. The inclusion and exclusion criteria are described in Table S6. From all subjects a signed informed consent was obtained. The research protocol was approved by the Medical Ethical Committee of the Academic Medical Centre of Amsterdam, the Netherlands and all methods were carried out in accordance with the approved guidelines. This study is registered at Current Controlled Trials (ISRCTN36043780). The date of registration was September 25^th^, 2013.

### Randomization, clinical examination and microbiological sampling

Randomization and allocation to the treatment groups (Test and Control) were achieved with a computer-generated sequence, that stratified for smoking habits. At the baseline appointment, every patient received an envelope containing either a prescription for a 0.12% chlorhexidine rinse (2 × day × 28 days) and antibiotics (amoxicillin 375 mg + metronidazole 250 mg, 3 times a day × 7 days, Test, *N *=* *18), or a prescription for only 0.12% chlorhexidine (Control, *N *=* *19).

At each appointment, plaque (presence/absence), bleeding on probing (BOP) (presence/absence), probing pocket depth (PPD, measure from the margin of the gingiva to the depth of the pocket) and clinical attachment loss (CAL, measured from the cemento-enamel junction or alternatively from the margin of a restoration to the bottom of the pocket) were recorded. For microbiological sampling, the deepest non-furcated site in each quadrant was selected. After supragingival plaque removal, 2 paper points (Henry Schein, Almere, The Netherlands) per site were inserted for 10 s up at the bottom of the pocket. The paper points from each individual were pooled and stored in a reduced transport medium^49^ at −80 °C until further analysis.

All clinical measurements and sampling were repeated at 3, 6 and 12 months after treatment and were performed by the same calibrated clinician.

### Periodontal therapy

The periodontal therapies were administered by 3 experienced dental hygienists from the Department of Periodontology at ACTA who were blinded to the treatment allocation. The treatment consisted of full-mouth supra- and subgingival scaling, root planing and oral hygiene instructions and was performed in 3 sessions within the same week. The patients were instructed to begin rinsing with chlorhexidine on the evening of the first treatment day. The patients in the Test group began with the first dose of antibiotics immediately before the first treatment session under the supervision of the researcher who performed the randomization. Four weeks after the last session of treatment, the patients were recalled and given supra- and subgingival scaling and oral hygiene checks. Thereafter, the patients followed a 3-monthly maintenance programme at the Department of Periodontology.

### Sample processing, 16S rRNA gene amplicon sequencing and data analysis

The paper points were submerged in 0.25 ml lysis buffer (LGC Genomics, Berlin, Germany), 0.2 ml roti-phenol (Carl Roth, Karlsruhe, Germany) and 0.25 ml of 0.1-mm zirconia beads (Lab Services BV, Breda, the Netherlands) and were beaten for 2 min with a Mini Beadbeater 96 (Biospec Products, Bartlesville, USA). DNA was extracted with the AGOWA mag Mini DNA Isolation Kit (LGC Genomics). In addition to the clinical samples, DNA extracted from duplicate sterile paper points and extraction blanks were included in all forthcoming steps to control for a potential contamination^45^. Barcoded amplicon libraries of the small subunit ribosomal RNA gene hypervariable region V5–V7 were generated for each of the individual samples as described previously^50^ and then pooled and sequenced by Macrogen Inc. (Seoul, Republic of Korea) using the 454 GS-FLX + Titanium system (Roche Molecular Diagnostics, Branford, CT, USA). The sequencing data were processed and filtered as described previously^44^. The sequences were clustered into operational taxonomic units (OTUs) and taxonomy was assigned as described previously^50^. The sequences are available at NCBI under the accession number PRJNA289294.

### Data analysis

The statistical analyses of the background and clinical characteristics of the study groups were performed with SPSS 20.0 (IBM Corporation, Armonk, New York, USA). The baseline differences between the Test and Control groups were analysed with Mann-Whitney-U test, independent T-test or Chi-square test. General linear models were used to test for changes in the clinical parameters within and between groups. repeated-measures analysis of variance (ANOVA), corrected for multiple testing (Bonferroni) was used to test for changes within the groups, and univariate analysis of covariance (ANCOVA) was used to test for changes between the groups at the different follow-up times. In the ANCOVAs, clinical parameters at each single evaluation were used as dependent variables, the corresponding parameters at baseline were used as covariates and treatment allocation was used as fixed factor.

A retrospective analysis of the responses to treatment was performed. The mean per cent changes in CAL (ΔCAL) at the four sampled sites for the whole study population at 12 months were compared with these values at baseline and the median was used as cut-off point. The individuals in the upper quantile (51^th^ – 100^th^ percentile) were arbitrarily termed “above median responders” (AMRs), and those in the lower quantile (0 – 50^th^ percentile) were termed “below median responders” (BMRs). Subsequent statistical analyses of the difference in the clinical effect of the treatment between the AMRs and BMRs were performed with repeated-measures ANOVA and ANCOVA as described above.

To allow for between-sample comparisons, the OTU-dataset was randomly subsampled at 1400 reads/sample. The Shannon diversity index, principal component analysis (PCA), one-way permutational multivariate analysis of variance (PERMANOVA) using the Bray-Curtis similarity measure and the Bray-Curtis similarity matrix itself were calculated in PAST version 3.0^51^. The data were log2 transformed for PCA analysis to normalize the distributions of taxa. The differences between treatments at the genus level were assessed using Mann-Whitney test, and the differences between the related samples (effects of time) were assessed with Wilcoxon signed ranks test in SPSS. The effect of the treatment group on Bray-Curtis similarity between the baseline and all follow-up samples of an individual were assessed using independent samples T-test. Changes in Shannon diversity within and between groups were tested with repeated-measures ANOVA and ANCOVA as described above. The relations between the Shannon Diversity Index and the per cent ΔCAL of the sampled sites and between the per cent ΔCAL of the samples sites and individual OTUs were assessed with Spearman correlation tests. The statistical tests for genus and the OTU analysis were not corrected for multiple testing. *P* < 0.05 was considered statistically significant

The assessments of the significance of the patterns of microbial co-occurrence or mutual exclusion at the genus or higher taxonomic level was performed using CoNet^52^. For this, a dataset of relative abundances of reads at the genus level including the 67 most abundant (average abundance 0.05% or above) genera or higher taxonomic level, and the remaining taxa collectively termed “Others”, was used (Table S7). An ensemble approach was used in which two measures of correlation (Pearson and Spearman) and two measures of dissimilarity (Bray-Curtis and Kullback-Leibler) were combined as described by Faust *et al*. (2012)^52^. The threshold for the correlations and the Bray-Curtis similarity was set at 0.6. The minimum number of methods that should support a link connecting two nodes was set at two. The data matrix was randomized by 100 row-wise permutations. The *P* values were adjusted with the Benjamini-Hochberg false discovery rate (FDR) correction for the number of tests, and only values of *P* < 0.05 were retained. The networks were visualized using Cytoscape v. 3.1.1^53^.

## Additional Information

**How to cite this article**: Bizzarro, S. *et al*. Microbial profiles at baseline and not the use of antibiotics determine the clinical outcome of the treatment of chronic periodontitis. *Sci. Rep*. **6**, 20205; doi: 10.1038/srep20205 (2016).

## Supplementary Material

Supplementary Information

Supplementary Table S1

## Figures and Tables

**Table 1:** Clinical parameters for the sampled sites at baseline and after treatment.

		Test	Control	*P*_*adj*_ value*
*N*		18	19	
PPD (mm)	Baseline	6.1 ± 1.2	6.2 ± 1.1	0.793
	3 months	3.8 ± 0.8^§^	4.4 ± 1.2^§^	0.040
	6 months	3.7 ± 0.6^§^	4.4 ± 1.0^§^	0.007
	12 months	3.6 ± 1.1^§^	4.4 ± 1.2^§^	0.035
CAL (mm)	Baseline	6.7 ± 2.0	6.5 ± 1.3	0.729
	3 months	5.2 ± 2.0^§^	5.4 ± 1.4^§^	0.084
	6 months	5.0 ± 2.0^§^	5.4 ± 1.3^§^	0.155
	12 months	4.9 ± 1.9^§^	5.3 ± 1.5^§^	0.331
BOP(%)	Baseline	95.8 ± 17.7	96.1 ± 9.4	0.963
	3 months	52.8 ± 39.2^§^	55.3 ± 35.9^§^	0.806
	6 months	58.3 ± 24.3^§^	55.3 ± 35.9^§^	0.756
	12 months	45.8 ± 36.6^§^	56.6 ± 33.2^§^	0.362
Plaque	Baseline	81.9 ± 25.4	67.1 ± 34.4	0.144
	3 months	45.8 ± 36.6^§^	31.6 ± 31.0^§^	0.510
	6 months	31.9 ± .7^§^	40.8 ± 31.4^§^	0.124
	12 months	38.9 ± 34.5^§^	35.5 ± 33.7^§^	0.704

PPD, probing pocket depth; CAL, clinical attachment level; BOP, bleeding on probing.

^*^Difference between Test and Control (ANCOVA adjusted for the corresponding variable at baseline). ^§^*P* < 0.05 in comparison with baseline (repeated-measures ANOVA). Values are means ± standard deviations.
