# Supplementary material for: Microbial profiles at baseline and not the use of antibiotics determine the clinical outcome of the treatment of chronic periodontitis
Source: Sci Rep. 2016 Feb 1;6:20205. doi: 10.1038/srep20205 (PMC4735321; doi:10.1038/srep20205)
Supplement: Supplementary Information [file srep20205-s1.pdf]

Microbial profiles at baseline and not the use of antibiotics determine the clinical outcome of treatment of chronic periodontitis

[1]S. Bizzarro*, [1]M.L. Laine, [2]M.J. Buijs, [2]B.W. Brandt, [2]W. Crielaard, [1]B.G. Loos, [2]E. Zaura

Supplementary Information

Figure S1 | Average Shannon Diversity Index per treatment group and time-point. Error bar indicates standard deviation. No statistically significant difference between the time-points (BL – baseline, 3M – 3 months, 6M – 6 months, 12M – 12 months) (*P*>0.05, repeated-measures ANOVA) and the treatments was found (*P*>0.05, ANCOVA).

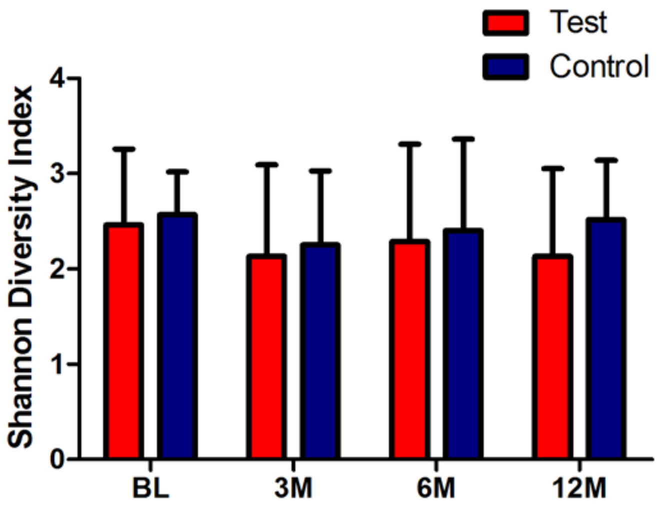

Figure S2 | Significant correlation between bacterial OTUs at baseline and treatment outcome measured % of Clinical Attachment Level change (% CAL change). R and *P* values are from Spearman correlation.

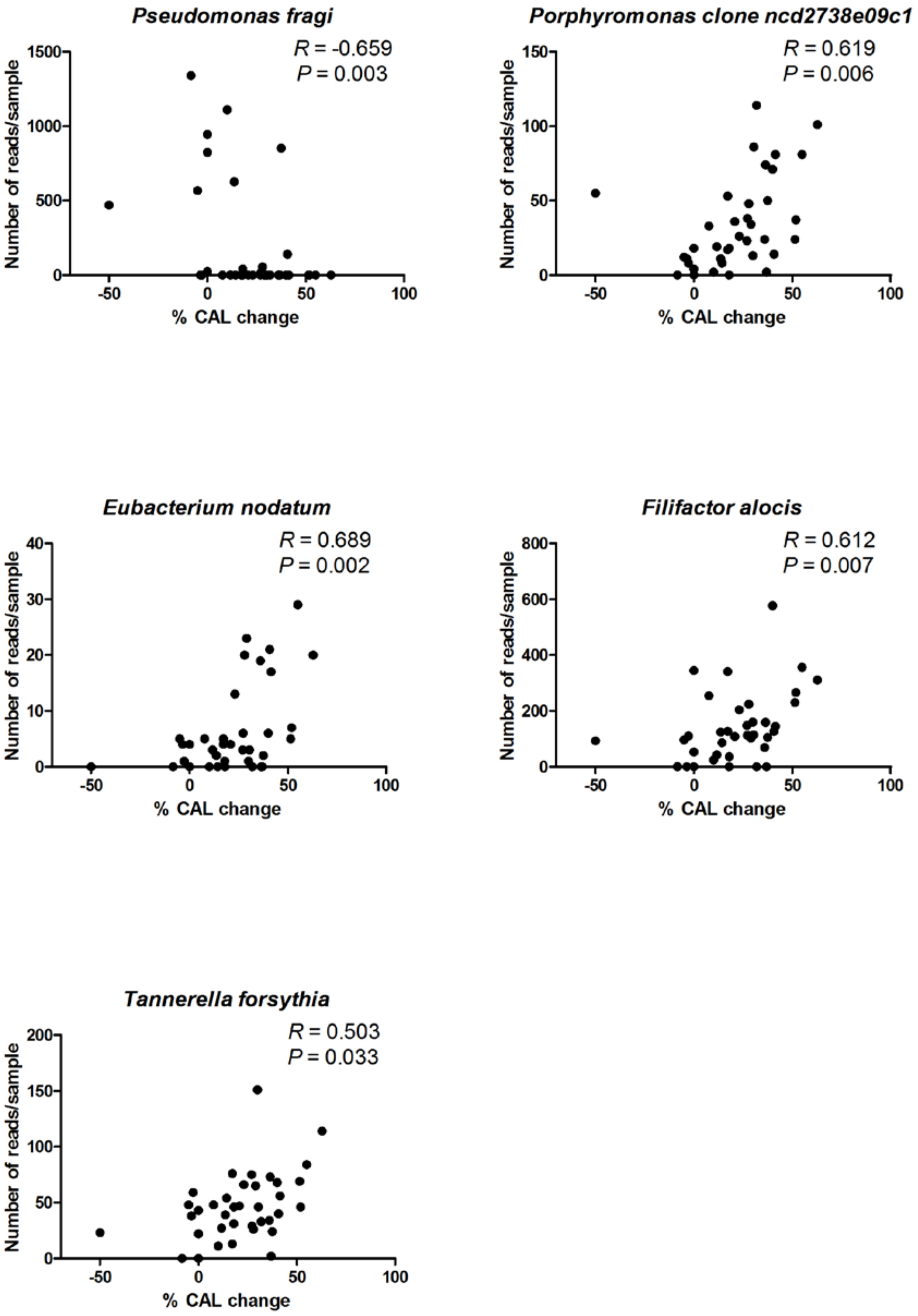

Table S1 | Background characteristics of the study groups at baseline.

		Test	Controls	*P* value
N		18	19	
Age (years)		48.1 ± 9.8	49.7 ± 6.2	NS
Gender	male	9 (50%)	13 (68%)	NS
Ethnicity	Dutch Caucasian	15 (84%)	16 (83%)	NS
Education	< high school	3 (17%)	2 (10%)	NS
	= high school	2 (11%)	3 (16%)	
	> high school	13 (72%)	14 (74%)	
Smoking	current	13 (68%)	8 (44%)	NS
	non smoker	6 (32%)	10 (56%)	

Values are means ± standard deviation or number of subjects (%). Independent T-test or χ^2 *P* values indicated.

Table S2 | Full-mouth clinical results after therapy.

		Test	Controls	P_{adj} value*
N		18	19	
PPD (mm)	Baseline	3.7 ± 0.6	3.9 ± 0.6	0.305
	3 months	2.7 ± 0.5§	3.0 ± 0.5§	0.142
	6 months	2.6 ± 0.5§	3.0 ± 0.5§	0.086
	12 months	2.7 ± 0.5§	3.0 ± 0.5§	0.199
nº PPD ≥5 mm	Baseline	50.5 ± 22.1	51.8 ± 22.9	0.850
	3 months	12.6 ± 10.2§	20.7 ± 16.9§	0.071
	6 months	11.6 ± 8.9§	20.8 ± 17.3§	0.037
	12 months	11.9 ± 10.4§	22.5 ± 20.7§	0.057
nº PPD ≥6 mm	Baseline	28.0 ± 17.4	34.9 ± 20.7	0.282
	3 months	3.6 ± 3.9§	10.5 ± 11.7§	0.047
	6 months	3.7 ± 3.9§	11.1 ± 12.9§	0.054
	12 months	4.1 ± 4.7§	11.7 ± 13.5§	0.057
nº PPD ≥7 mm	Baseline	18.0 ± 13.2	20.0 ± 15.9	0.679
	3 months	0.8 ± 1.5§	4.7 ± 6.2§	0.013
	6 months	1.2 ± 2.1§	5.3 ± 6.4§	0.013
	12 months	0.7 ± 1.1§	5.4 ± 7.6§	0.016
CAL (mm)	Baseline	4.0 ± 1.1	4.4 ± 0.8	0.186
	3 months	3.3 ± 1.2§	3.9 ± 0.8§	0.297
	6 months	3.3 ± 1.1§	3.9 ± 0.8§	0.300
	12 months	3.3 ± 1.2§	3.9 ± 0.8§	0.438
BOP (%)	Baseline	69.5 ± 16.3	64.9 ± 13.3	0.349
	3 months	20.7 ± 11.9§	20.1 ± 11.8§	0.945
	6 months	20.0 ± 14.8§	22.5 ± 14.9§	0.512
	12 months	21.5 ±14.7§	22.0 ± 12.8§	0.825
Plaque (%)	Baseline	69.1 ± 25.1	56.6 ± 27.7	0.159
	3 months	17.3 ± 14.9§	17.6 ± 18.3§	0.348
	6 months	17.0 ± 18.5§	22.0 ± 21.3§	0.169
	12 months	19.4 ± 15.1§	19.5 ± 15.3§	0.679

PPD, probing pocket depth; CAL, clinical attachment level; BOP, bleeding on probing. Values are means ± standard deviations. *Difference between Test and Control (ANCOVA, adjusted for the corresponding variable at baseline). §*P* <0.05 in comparison with baseline (repeated-measures ANOVA)

Table S3 | Classification of reads at genus level or higher taxon.

Genus	Test	Control
Fusobacterium	24.00 ± 18.11	22.11 ± 13.77
Uncl. Pseudomonodaceae	13.24 ± 30.46	13.86 ± 23.01
Filifactor	8.88 ± 8.03	11.33 ± 9.96
Treponema	5.67 ± 7.18	6.60 ± 6.49
Paludibacter	4.98 ± 5.66	7.18 ± 8.55
Porphyromonas	4.40 ± 5.60	4.45 ± 3.94
Tannerella	3.01 ± 1.87	3.69 ± 3.69
Clostridiales Fam.xiii IS	3.10 ± 2.27	2.86 ± 2.17
Prevotella	3.52 ±4.38	4.28 ± 4.21
Acinetobacter	2.73 ± 7.54	0.12 ± 0.25
Parvimonas	2.86 ± 2.33	2.57 ± 2.71
Synergistaceae	2.17 ± 1.51	2.79 ± 1.70
Actinomyces	1.63 ± 2.12	1.41 ± 1.28
Streptococcus	0.68 ± 0.66	0.76 ± 1.34
Neisseria	0.96 ±1.05	1.11 ± 2.91
Campylobacter	0.73 ± 0.54	1.17 ± 1.21
Rothia	0.65 ± 1.02	0.59 ± 1.16
Capnocytophaga	0.60 ± 0.95	0.13 ± 0.19
Veillonella	0.38 ± 0.49	0.80 ± 1.35
Haemophilus	0.24 ±0.37	0.38 ± 1.06
Micrococcales	0.07 ± 0.17	0.12 ± 0.46
Gemella	0.40 ± 0.61	0.26 ± 0.32
Corynbacterium	0.35 ± 0.48	0.23 ± 0.49
Selenomonas	0.69 ±0.70	0.84 ± 0.98
Peptostreptococcus Fam. IS	0.33 ± 0.31	0.72 ± 1.11
Wchb1-69	0.52 ± 0.71	1.04 ± 2.09
Leptotrichia	0.35 ± 0.48	0.13 ± 0.22
Johnsonella	0.48 ± 0.55	0.82 ± 1.11
Derxia	0.10 ± 0.14	0.08 ± 0.35
Aggregatibacter	0.85 ± 2.40	0.03 ± 0.09

Reads account for 90% of all OTUs at baseline for Test and Control. Values indicate relative abundance (mean ± standard deviation).

Table S4 | Full mouth clinical parameters at baseline and after treatment according to the response to therapy.

		BMR	AMR	P_{adj} value*
N		19	18	
CAL (mm)	Baseline	4.4 ± 1.0	3.9 ± 0.9	0.139
	3 months	4.0 ± 1.1§	3.1 ± 0.8§	0.003
	6 months	4.0 ± 1.1§	3.1 ± 0.9§	0.002
	12 months	4.1 ± 1.1	3.0 ± 0.8§	<0.001
PPD (mm)	Baseline	3.9 ± 0.6	3.8 ± 0.6	0.785
	3 months	3.1 ± 0.6§	2.6 ± 0.3§	0.001
	6 months	3.1 ± 0.6§	2.6 ± 0.3§	<0.001
	12 months	3.1 ± 0.6§	2.5 ± 0.3§	<0.001
BOP (%)	Baseline	66.7 ± 16.8	67.6 ± 12.8	0.869
	3 months	23.2 ±12.5§	17.4 ± 10.3§	0.115
	6 months	26.7 ± 17.2§	15.5 ± 8.7§	0.017
	12 months	25.2 ± 16.9§	21.3 ± 14.7§	0.143
Plaque (%)	Baseline	62.1 ± 23.4	63.2 ± 30.8	0.899
	3 months	15.7 ± 15.4§	19.2 ± 17.8§	0.511
	6 months	23.9 ± 23.1§	15.0 ± 15.2§	0.137
	12 months	22.5 ± 16.2§	16.2 ± 13.5§	0.187

BMR, below median responders; AMR, above median responders. Values are means ± standard deviations.

*Difference between groups (ANCOVA). *P* values are adjusted for the corresponding baseline variable. §*P* <0.05 in comparison with baseline (repeated-measures ANOVA).

**Table S5 | Association between presence of taxa with treatment outcome per treatment group at the different time visits.**				
	**Test**		**Control**	
**time visit**	**BMR**	**AMR**	**BMR**	**AMR**
Baseline		*Porphyromonas*	-	-
3 months		*Actinomyces*<br>*Haemophilus*		*Haemophilus*<br>*Rothia*<br>*Streptococcus*
6 months			*Paludibacter*<br>*Tannerella*<br>*Clostridiales fam xiii is*<br>*Treponema*<br>*Unc. Synergistaceae*<br>*Filifactor*	
12 months		*Capnocytophaga*	*Paludibacter*<br>*Tannerella*<br>*Unc. Synergistaceae*	*Rothia*

BMR= below median responders. AMR= above median responders. Taxa, which show significant higher abundance between BMR and AMR, are represented.

**Table S6 | Inclusion/exclusion criteria**	
Inclusion	2 teeth per quadrant with pocket ≥5 mm with clinical evidence of attachment loss
	>50% bleeding on probing
	≥20 teeth present
Exclusion	Any systemic disease
	Chronic use of medications
	Use of systemic antibiotics in the last 6 months
	Previous periodontal treatment in the last 2 years
	Pregnancy/lactation
	Presence of implants or orthodontic appliances

**Table S7 | Dataset of relative abundances of reads used for microbial co-occurrence or mutual exclusion.**				
**Genus or higher taxon**	**BMR**		**AMR**	
	**Test**	**Control**	**Test**	**Control**
Fusobacterium	0.2534 ± 0.2091	0.2357 ± 0.1387	0.2265 ± 0.1599	0.2049 ± 0.1430
Pseudomonadaceae	0.2633 ± 0.3983	0.1882 ± 0.2541	0.0017 ± 0.0042	0.0834 ± 0.2000
Prevotella	0.0252 ± 0.0327	0.0378 ± 0.0358	0.0451 ± 0.0528	0.0484 ± 0.0499
Paludibacter	0.0715 ± 0,0728	0.0911± 0,1081	0.0280 ± 0,0213	0.0502 ± 0.0484
Acinetobacter	0.0247 ± 0.0719	0.0016 ± 0.0032	0.0299 ± 0.0831	0.0008 ± 0.0013
Neisseria	0.0121 ± 0.0128	0.0052 ± 0.0060	0.0071 ± 0.0076	0.0176 ± 0.0422
Filifactor	0.0669 ± 0.0805	0.0859 ± 0.0765	0.1108 ± 0.0783	0.1437 ± 0.1172
Actinomyces	0.0219 ± 0.0286	0.0143 ± 0.0132	0.0106 ± 0.0082	0.0139 ± 0.0132
Rothia	0.0097 ± 0.0123	0.0061 ± 0.0132	0.0033 ± 0.0072	0.0057 ± 0.0104
Treponema	0.0296 ± 0.0322	0.0454 ± 0.0490	0.0838 ± 0.0910	0.0889 ± 0.0754
Parvimonas	0.0286 ± 0.0226	0.0300 ± 0.0295	0.0287 ± 0.0254	0.0209 ± 0.0249
Porphyromonas	0.0142 ± 0.0139	0.0402 ± 0.0502	0.0737 ± 0.0670	0.0493 ± 0.0248
Streptococcus	0.0071 ± 0.0068	0.0086 ± 0.0146	0.0065 ± 0.0069	0.0063 ± 0.0128
Veillonella	0.0036 ± 0.0055	0.0110 ± 0.0169	0.0040 ± 0.0046	0.0048 ± 0.0080
Clostridiales Fam xiii IS	0.0273 ± 0.0233	0.0284 ± 0.0188	0.0517 ± 0.0267	0.0441 ± 0.0307
Tannerella	0.0260 ± 0.0201	0.0269 ± 0.0113	0.0342 ± 0.0174	0.0481 ± 0.0308
Capnocytophaga	0.0066 ± 0.0118	0.0016 ± 0.0023	0.053 ± 0.0072	0.0010 ± 0.0015
Unc Synergistaceae	0.0194 ± 0.0152	0.0245 ± 0.0201	0.0240 ± 0.0155	0.0316 ± 0.0131

Haemophilus	0.0016 ± 0.0030	0.0025 ± 0.0062	0.0032 ± 0.0043	0.0052 ± 0.0144
Campylobacter	0.0058 ± 0.0049	0.0125 ± 0.0147	0.0089 ± 0.0056	0.0108 ± 0.0094
Micrococcales	0.0008 ± 0.0019	0.0003 ± 0.0006	0.0006 ± 0.0017	0.0023 ± 0.0066
Gemella	0.0032 ± 0.0072	0.0033 ± 0.0037	0.0049 ± 0.0054	0.0017 ± 0.0025
Corynebacterium	0.0037 ± 0.0064	0.0030 ± 0.0064	0.0034 ± 0.0029	0.0016 ± 0.0026
Selenomonas	0.0048 ± 0.0054	0.0086 ± 0.0108	0.0092 ± 0.0082	0.0083 ± 0.0092
Peptostreptococcaceae IS	0.0036 ± 0.0035	0.0071 ± 0.0140	0.0031 ± 0.0029	0.0075 ± 0.0094
Sphingobacteriales Wchb1-69	0.0028 ± 0.0059	0.0028 ± 0.0036	0.0076 ± 0.0077	0.0190 ± 0.0286
Johnsonella	0.0037 ± 0.0056	0.0065 ± 0.0119	0.0059 ± 0.0055	0.0102 ± 0.0105
Leptotrichia	0.0022 ± 0.0036	0.0016 ± 0.0026	0.0048 ± 0.0056	0.0011 ± 0.0018
Derxia	0.0012 ± 0.0017	0.0001 ± 0.0002	0.0010 ± 0.0013	0.0017 ± 0.0052
Aggregatibacter	0.0062 ± 0.0165	0.0001 ± 0.0003	0.0108 ± 0.0300	0.0006 ± 0.0013
Dialister	0.0017 ± 0.0023	0.0062 ± 0.0116	0.0030 ± 0.0033	0.0013 ± 0.0015
Pasteurellaceae	0.0021 ± 0.0035	0.0014 ± 0.0022	0.0045 ± 0.0072	0.0036 ± 0.0039
Burkholderia	0.0015 ± 0.0045	0.0036 ± 0.0113	0.0486 ± 0.1204	0.0007 ± 0.0021
Candidate division tm7	0.0038 ± 0.0052	0.0055 ± 0.0072	0.0036 ± 0.0041	0.0034 ± 0.0050
Veillonellaceae	0.0024 ± 0.0035	0.0030 ± 0.0057	0.0052 ± 0.0067	0.0028 ± 0.0029
Anaeroglobus	0.0021 ± 0.0038	0.0025 ± 0.0041	0.0010 ± 0.0024	0.0015 ± 0.0026
Bacteria	0.0019 ± 0.0033	0.0025 ± 0.0031	0.0054 ± 0.0100	0.0047 ± 0.0027
Mycoplasma	0.0017 ± 0.0029	0.0034 ± 0.0075	0.0024 ± 0.0032	0.0034 ± 0.0048
Desulfobulbus	0.0055 ± 0.0060	0.0037 ± 0.0026	0.0057 ± 0.0048	0.0053 ± 0.0026
Firmicutes	0.0050 ± 0.0058	0.0031 ± 0.0038	0.0057 ± 0.0063	0.0046 ± 0.0036

Bulleidia	0.0013 ± 0.0015	0.0044 ± 0.0064	0.0005 ± 0.0010	0.0028 ± 0.0039
Leptotrichiaceae	0.0003 ± 0.0006	0.0020 ± 0.0058	0.0047 ± 0.0093	0.0011 ± 0.0033
Propionibacteriaceae	0.0006 ± 0.0012	0	0.0005 ± 0.0009	0.0002 ± 0.0007
Brevundimonas	0.0006 ± 0.0017	0.0003 ± 0.0009	0.0010 ± 0.0029	0
Mogibacterium	0.0040 ± 0.0079	0.0031 ± 0.0030	0.0029 ± 0.0029	0.0028 ± 0.0026
Prevotellaceae	0.0006 ± 0.0008	0.0009 ± 0.0011	0.0013 ± 0.0016	0.0038 ± 0.0055
Peptococcus	0.0006 ± 0.0006	0.0004 ± 0.0008	0.0037 ± 0.0045	0.0011 ± 0.0013
Rikenellaceae Rc9 gut group	0.0010 ± 0.0016	0.0016 ± 0.0018	0.0011 ± 0.0019	0.0052 ± 0.056
Catonella	0.0005 ± 0.0012	0.0016 ± 0.0029	0.0025 ± 0.0024	0.0026 ± 0.0052
Actinobaculum	0.0006 ± 0.0017	0	0.0021 ± 0.0028	0.0004 ± 0.0005
Cardiobacterium	0.0006 ± 0.0008	0.0002 ± 0.0003	0.0013 ± 0.0023	0.0003 ± 0.010
Abiotrophia	0.0001 ± 0.0002	0	0.0002 ± 0.0005	0
Micrococcus	0.0002± 0.0007	0	0.0007 ± 0.0014	0
Oribacterium	0.0011 ± 0.0023	0.0021 ± 0.0031	0.0003 ± 0.0007	0.0020 ± 0.0054
Xylanibacter	0.0001± 0.0002	0.0009 ± 0.0018	0.0090 ± 0.0215	0.0018 ± 0.0028
Bergeyella	0.0002 ± 0.0005	0.0001 ± 0.0002	0.0007 ± 0.0019	0.0006 ± 0.0011
Desulfohalobiaceae	0	0.0003 ± 0.0009	0.0130 ± 0.0390	0
Peptostreptococcus	0.0005 ± 0.0012	0.0010 ± 0.0029	0.0014 ± 0.0021	0.0015 ± 0.0045
Atopobium	0.0006 ± 0.0009	0.0008 ± 0.0012	0.0004 ± 0.0007	0
Phocaeicola	0.0006 ± 0.0014	0.0009 ± 0.0013	0.0010 ± 0.0018	0.0032 ± 0.0053
Solobacterium	0.0004 ± 0.0010	0.0007 ± 0.0018	0	0.0002 ± 0.0003
Odoribacter	0	0.0061 ± 0.0192	0.0025 ± 0.0052	0

Brachybacterium	0.0001 ± 0.0002	0	0.0017 ± 0.0050	0
Clostridiales Fam xi IS	0.0006 ± 0.0019	0.0023 ± 0.0050	0.0037 ± 0.0067	0.0002 ± 0.0005
Rhizobiales	0.0001 ± 0.0002	0.0001 ± 0.0002	0.0029 ± 0.0088	0.0000 ± 0.0000
Enhydrobacter	0.0001 ± 0.0002	0.0001 ± 0.0002	0	0.0002 ± 0.0003
Neisseriaceae	0.0002 ± 0.0004	0.0001 ± 0.0002	0.0003 ± 0.0005	0.0001 ± 0.0002
Others	0.0062 ± 0.0049	0.0043 ± 0.0050	0.0171 ± 0.0152	0.0050 ± 0.0056

BMR, below median responders; AMR, above median responders. Values are means ± standard deviations.
